# Overexpression of PVR and PD-L1 and its association with prognosis in surgically resected squamous cell lung carcinoma

**DOI:** 10.1038/s41598-021-87624-x

**Published:** 2021-04-20

**Authors:** Jii Bum Lee, Min Hee Hong, Seong Yong Park, Sehyun Chae, Daehee Hwang, Sang-Jun Ha, Hyo Sup Shim, Hye Ryun Kim

**Affiliations:** 1 Division of Medical Oncology, Department of Internal Medicine, Yonsei Cancer Center, Yonsei University College of Medicine, 50-1 Yonsei-ro, Seodaemun-gu, Seoul, Republic of Korea; 2 Division of Hemato-oncology, Wonju Severance Christian Hospital, Yonsei University College of Medicine, Wonju, Republic of Korea; 3 Department of Thoracic and Cardiovascular Surgery, Yonsei University College of Medicine, Seoul, Korea; 4 Korea Brain Bank, Korea Brain Research Institute, Daegu, Korea; 5 Department of Biological Sciences, Seoul National University, Seoul, Korea; 6 Department of Biochemistry, College of Life Science & Biotechnology, Yonsei University, Seoul, Korea; 7 Department of Pathology, Yonsei University College of Medicine, 50-1 Yonsei-ro, Seodaemun-gu, Seoul, Republic of Korea

**Keywords:** Lung cancer, Tumour biomarkers

## Abstract

Targeting T-Cell Immunoreceptor with Ig and ITIM domain-poliovirus receptor (PVR) pathway is a potential therapeutic strategy in lung cancer. We analyzed the expression of PVR and programmed death ligand-1 (PD-L1) in surgically resected squamous cell lung carcinoma (SQCC) and determined its prognostic significance. We collected archival surgical specimens and data of 259 patients with SQCC at Yonsei Cancer Center (1998–2020). Analysis of variance was used to analyze the correlations between PVR and PD-L1 expression and patient characteristics. Kaplan–Meier curves were used to estimate recurrence-free survival (RFS) and overall survival (OS). Most patients were male (93%); the majority were diagnosed with stage 1 (47%), followed by stage 2 (29%) and stage 3 (21%). Overexpression of PVR resulted in a significantly shorter median RFS and OS (*P* = 0.01). PD-L1 expression was not significant in terms of prognosis. Patients were subdivided into four groups based on low and high PVR and PD-L1 expression. Those expressing high levels of PVR and PD-L1 had the shortest RFS (*P* = 0.03). PVR overexpression is associated with a poor prognosis in surgically resected SQCC. Inhibition of PVR as well as PD-L1 may help overcome the lack of response to immune checkpoint monotherapy.

## Introduction

Squamous cell lung carcinoma (SQCC) accounts for 20–30% of all non-small cell lung cancers (NSCLCs)^1^. With distinct clinicopathological features, such as a lack of targetable mutations, higher incidence in older individuals, and advanced or metastatic disease at diagnosis, patients with SQCC have a shorter lifespan than those with other NSCLC subtypes^2–4^. Despite surgical resection, many patients with NSCLC experience relapse^5^. In surgically resected NSCLC (pathological stages IA–IIB), the 5-year survival rate is approximately 40–70%, but drops to 13% and 5% for stage IIIA and IIIB disease, respectively^6^.

Prior to immunotherapy, the first-line treatment options for advanced SQCC were limited to platinum-doublet chemotherapy, with a median survival of 8–11 months^7^. The advent of pembrolizumab, a programmed cell death protein-1 (PD-1) inhibitor, revolutionized the first-line treatment options for both patients with squamous and non-squamous NSCLC with programmed death ligand-1 (PD-L1) expression levels of ≥ 50%^8^. However, only 30% of patients with advanced NSCLC exhibit high levels of PD-L1 expression and are eligible for treatment with pembrolizumab^8,9^.

Recently, adding pembrolizumab to chemotherapy resulted in a significant survival advantage for patients with advanced SQCC in first-line settings, regardless of PD-L1 expression^10^. Although the PD-L1 tumor proportion score is currently a biomarker for first-line treatment using pembrolizumab, the predictive role of PD-L1 in combination treatment needs further validation since patients across all categories of PD-L1 expression showed improved outcomes with combination treatment. Other combination treatments with immune checkpoint inhibitors—such as ipilimumab, a monoclonal antibody against cytotoxic T-lymphocyte-associated protein-4—and chemotherapy did not improve overall survival (OS) in patients with metastatic squamous or non-squamous NSCLC^11^.

Other checkpoint receptor blockers that modulate immune cell activation are currently under investigation since a significant proportion of patients do not respond to immunotherapy^12^. Among checkpoint blockers, T-cell immunoglobulin and ITIM domain (TIGIT) binds to poliovirus receptor (PVR). TIGIT is enriched on T-cells, such as regulatory T-cells, type 1 regulatory T-cells, memory T-cells, exhausted CD8 + T-cells, natural killer T-cells, and natural killer cells^13,14^. Mouse models have shown that TIGIT/PVR binding induces immune evasion of tumor cells, one of the key hallmarks of cancer^15,16^. Levels of PVR are usually low (or PVR is not expressed) in normal tissues, but are high in tumor cells. PVR expression is associated with the invasion, migration, and proliferation of tumor cells^17^. Overexpression of PVR is observed in multiple malignancies, and tumors expressing high levels of PVR are also associated with poor prognosis^18–20^.

Recently, several clinical trials have evaluated the efficacy of blocking the TIGIT/PVR axis in solid cancers such as NSCLC^21^. Tiragolumab, an anti-TIGIT antibody, in combination with atezolizumab, an anti-PD-L1 agent, showed a promising treatment response and a manageable toxicity profile in first-line NSCLC treatment. Compared to monotherapy (atezolizumab), combination therapy improved the objective response rate (31.3% *vs.* 16.2%) and median progression-free survival (5.4 *vs.* 3.6 months). These results strongly suggest that the TIGIT/PVR axis may be a clinically useful target for treating patients with NSCLC.

**Figure 1:**
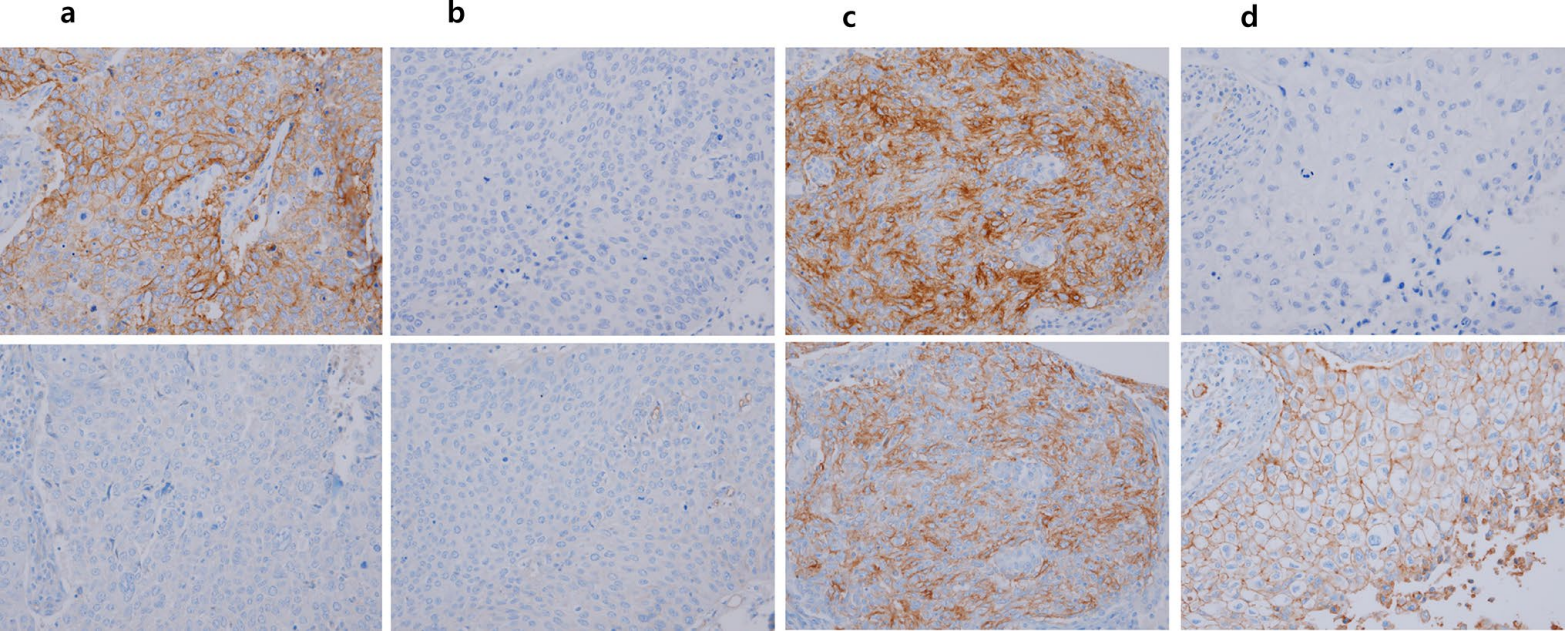
Programmed death ligand-1 (PD-L1) and poliovirus receptor (PVR) expression analysis using immunohistochemistry with ×200 magnification. (**a**) PD-L1^hi^/PVR^lo^, (**b**) PD-L1^lo^/PVR^lo^, (**c**) PD-L1^hi^/PVR^hi^, and (**d**) PD-L1^lo^/PVR^hi^.

Despite increasing evidence of the involvement of the TIGIT/PVR axis, few studies have addressed the prognostic role of PVR^22^. Therefore, we aimed to assess PVR expression in surgically resected SQCC tissues to determine its relationship with PD-L1. We evaluated the correlations of PVR and PD-L1 expression with clinicopathological factors and determined their prognostic implications for survival outcomes in SQCC (Fig. 1).

## Results

### PVR expression in SQCC (The Cancer Genome Atlas [TCGA] data)

**Figure 2:**
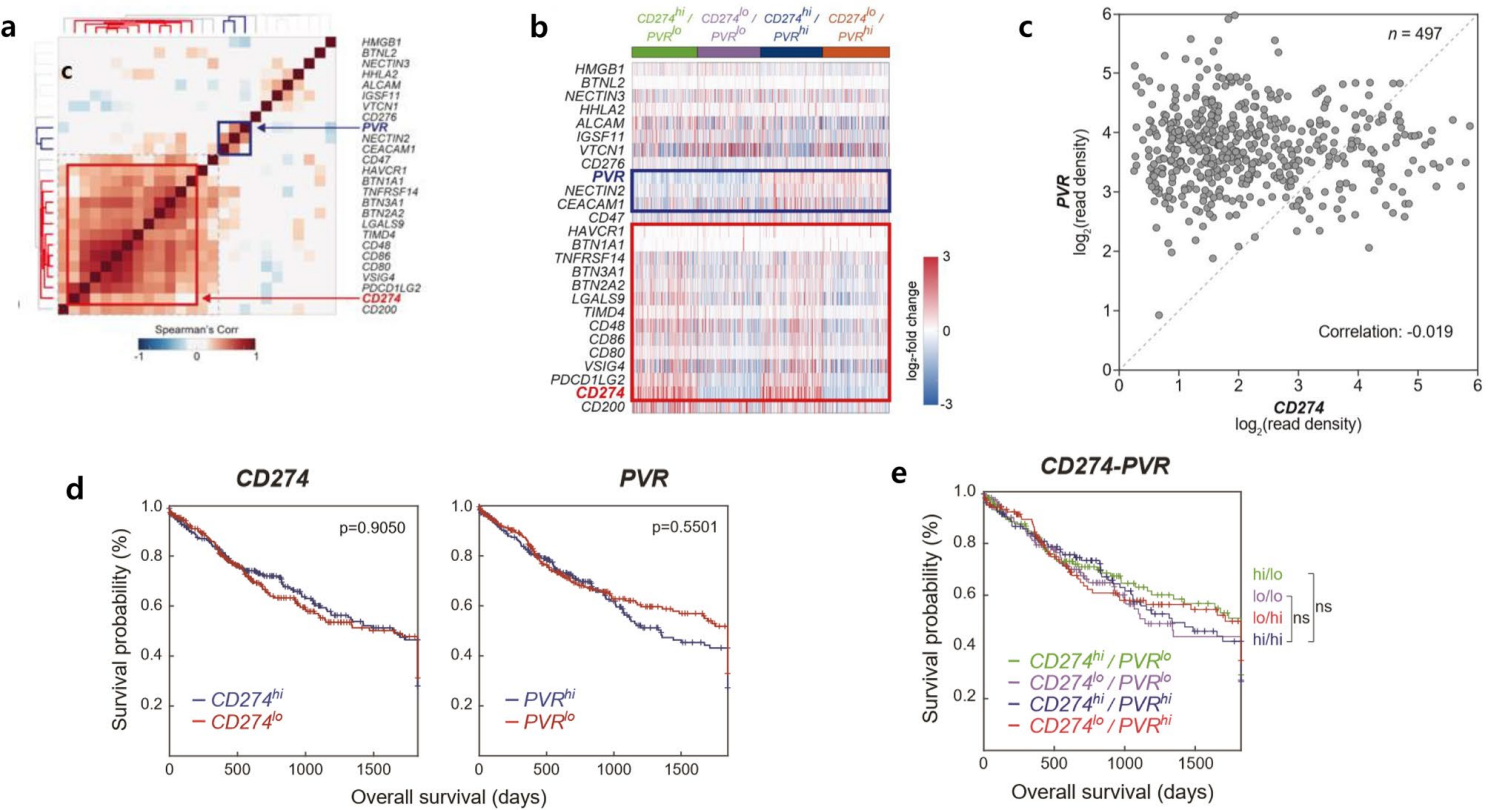
Co-expression patterns of immune checkpoint ligands (ICLs) and immune checkpoint receptors in The Cancer Genome Atlas data of lung squamous cell carcinoma. (**a**) Heatmap showing Spearman’s correlation coefficients for all pairs of ICLs. The dendrogram shows the results of hierarchical clustering of ICLs based on the correlation coefficients using Euclidean distance as a dissimilarity measure and the single linkage method. Red boxes represent programmed death ligand-1 (PD-L1/CD274); blue boxes represent poliovirus receptor (PVR). (**b**) Gene expression patterns of ICLs among four patient groups: (1) PD-L1^hi^/PVR^lo^, (2) PD-L1^lo^/PVR^lo^, (3) PD-L1^hi^/PVR^hi^, and (4) PD-L1^lo^/PVR^hi^. Red represents increased median expression and blue represents decreased median expression levels of each ICL. The colored bar shows the gradient of log_2_ fold-changes in expression levels. (**c**) Scatter plot showing patterns of PD-L1 and PVR. (**d**) Kaplan–Meier analysis of overall survival according to high and low PD-L1 (CD274) and PVR expression, respectively. (**e**) Multivariate analyses of four patient groups show no statistically significant differences between the groups.

We analyzed TCGA RNA-sequencing data of gene expression profiles in SQCC to identify the co-expression patterns of immune checkpoint ligands (ICLs)^23^. Figure 2a, b consists of two main clusters, including the PD-L1 (CD274) cluster of 10 other ICLs (CD48/80/86, BTN2A2/3A1, TIMD4, VSIG4, PDCD1LG2, TNFRSF14, and LGALS9) and the PVR cluster of PVR, NECTIN2, and CD276. Clustering analysis showed no correlation between the PD-L1 (CD274) cluster (red boxes) and the PVR cluster (blue boxes). Scatter plot also showed no correlation between the PD-L1 (CD274) cluster and the PVR cluster (Fig. 2c).

According to the TCGA data, PVR was expressed independently of PD-L1 expression in SQCC. We also analyzed the survival difference for both high expression and low expression of PD-L1 and PVR but found no significant differences in OS between the groups with high and low PD-L1 (*P* = 0.91) and PVR expression (*P* = 0.55; Fig. 2d). We further categorized patients into four groups depending on PD-L1 and PVR expression: PD-L1^hi^/PVR^lo^, PD-L1^lo^/PVR^lo^, PD-L1^hi^/PVR^hi^, and PD-L1^lo^/PVR^hi^ (Fig. 2e). Similarly, no significant differences were found between the four subgroups using multivariate analyses. There was a trend for the PD-L1^hi^/PVR^hi^ group to have the worst survival rate, indicating that high expression of PD-L1 and PVR may contribute to poor prognosis.

### Baseline clinicopathological characteristics

In total, 259 patients were included (see Supplementary Table S1 online). Patients were distributed evenly across the age groups (< 65 years, 47%; ≥ 65 years, 53%). Most patients were male (93%, *n* = 241). When classified according to American Joint Committee on Cancer (seventh edition) stage, the majority of patients were initially diagnosed with stage 1 disease (47%, *n* = 122), followed by stage 2 (29%, *n* = 76) and stage 3 disease (21%, *n* = 54). Pleural involvement and lymphovascular invasion were observed in 26% (*n* = 68) and 11% (*n* = 28) of patients, respectively. Most patients had a history of smoking, with 41% (*n* = 106) being smokers at the time of hospitalization; 44% (*n* = 114) were former smokers. Adjuvant chemotherapy and radiotherapy were administered to 85% (*n* = 221) and 17% (*n* = 44) of patients, respectively.

Table 1 shows no significant differences in age, sex, pathological T/N stage, EGFR and KRAS mutation, pleural involvement, lymphovascular invasion, smoking status, and adjuvant chemotherapy and radiotherapy among the different groups (grouped according to PD-L1 and PVR expression). The distribution of PD-L1, PVR, and CD8^+^ expression is depicted in Supplementary Fig. S3 online.

**Table 1:** Comparison of clinical characteristics between patients based on PVR and PD-L1 expression levels.

Clinical characteristic	PD-L1^hi^/PVR^lo^		PD-L1^lo^/PVR^lo^		PD-L1^hi^/PVR^hi^		PD-L1^lo^/PVR^hi^		*P*-value
	*n*	%	*n*	%	*n*	%	*n*	%	
Number of patients	37	14	95	37	33	13	94	36	
Age, years									0.99
< 65	18	49	45	47	15	45	45	48	
≥ 65	19	51	50	53	18	55	49	52	
Sex									0.91
Male	35	95	87	92	31	94	88	94	
Female	2	5	8	8	2	6	6	6	
Pathological T stage									0.36
T1	2	5	19	20	5	15	9	10	
T2	29	78	63	66	21	64	62	66	
T3	5	14	11	12	6	18	19	20	
T4	1	3	2	2	1	3	4	4	
Pathological N stage									0.48
N0	17	46	57	60	21	64	47	50	
N1	15	40	22	23	5	15	29	31	
N2	5	14	15	16	7	21	17	18	
N3	0	0	1	1	0	0	1	1	
AJCC stage (seventh edition)									0.38
I	15	40	49	52	16	48	42	45	
II	15	40	28	29	9	28	24	26	
IIIA	5	14	17	18	7	21	25	27	
IIIB	1	3	1	1	1	3	3	2	
IV	1	3	0	0	0	0	0	0	
Pleural involvement									0.63
Positive	8	22	23	24	8	24	29	31	
Negative	29	78	72	76	25	76	65	69	
Lymphovascular invasion									0.76
Positive	3	8	9	9	5	16	11	12	
Negative	34	92	86	91	28	84	83	88	
*EGFR* mutation									
Yes	0	0	2	2	0	0	2	2	0.31
No	16	43	21	22	16	48	15	16	
Unknown	21	57	72	76	17	52	77	82	
*KRAS* mutation									
Yes	0	0	0	0	0	0	0	0	0.31
No	14	38	22	23	15	45	17	18	
Unknown	23	62	73	77	18	55	77	82	
Smoking status									0.58
Never-smoker	7	19	15	16	2	6	15	16	
Former smoker	16	43	46	48	14	42	38	40	
Current smoker	14	38	34	36	17	52	41	44	
Adjuvant therapy									
Adjuvant chemotherapy									0.69
Yes	33	89	78	82	29	88	81	86	
No	4	11	17	18	4	12	13	14	
Adjuvant radiotherapy									0.36
Yes	6	16	13	14	4	12	21	22	
No	31	84	82	86	29	88	73	78	

AJCC, American Joint Committee on Cancer; PD-L1, programmed death ligand-1; PVR, poliovirus receptor; *hi*, high; *lo*, low.

### Association between PVR expression and survival

**Figure 3:**
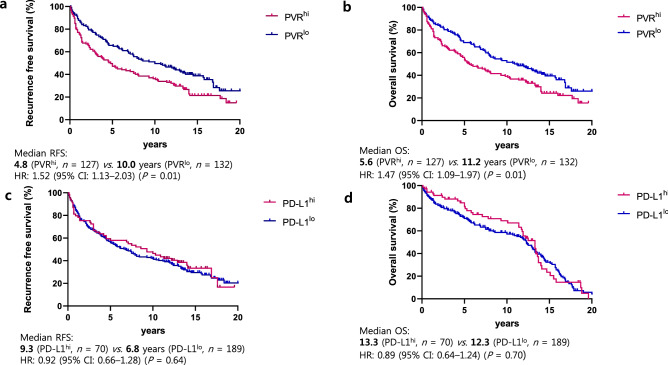
Kaplan–Meier analysis of recurrence-free survival (RFS) and overall survival (OS) by poliovirus receptor (PVR) and programmed death ligand-1 (PD-L1) expression. HR, hazard ratio; CI, confidence interval.

In conjunction with the survival curves drawn using TCGA data of lung squamous cell carcinoma (Fig. 2c), we analyzed the median recurrence-free survival (mRFS) and median OS (mOS) of patients according to high or low expression levels of PD-L1 (CD274) and PVR (Fig. 3). High PVR expression was associated with inferior mRFS and mOS. The mRFS was 4.8 months for high PVR expression and 10.0 years for low PVR expression (hazard ratio [HR]: 1.54, 95% confidence interval [CI]: 1.15–2.07; *P* = 0.01) (Fig. 3a). The corresponding mOS values were 5.6 and 11.2 years, respectively (HR: 1.45, 95% CI: 1.10–2.00; *P* = 0.01) (Fig. 3b). In contrast, high PD-L1 expression or high CD8^+^ expression was not associated with either poor mRFS or mOS (Fig. 3c, d and see Supplementary Fig. S4 online).

### Association of PVR and PD-L1 expression with clinical characteristics

Patients were further categorized into four groups depending on the expression levels of both PD-L1 and PVR, based on the median expression level: PD-L1^hi^/PVR^lo^ (*n* = 37, 14%), PD-L1^lo^/PVR^lo^ (*n* = 95, 37%), PD-L1^hi^/PVR^hi^ (*n* = 33, 13%), and PD-L1^lo^/PVR^hi^ (*n* = 94, 36%).

**Figure 4:**
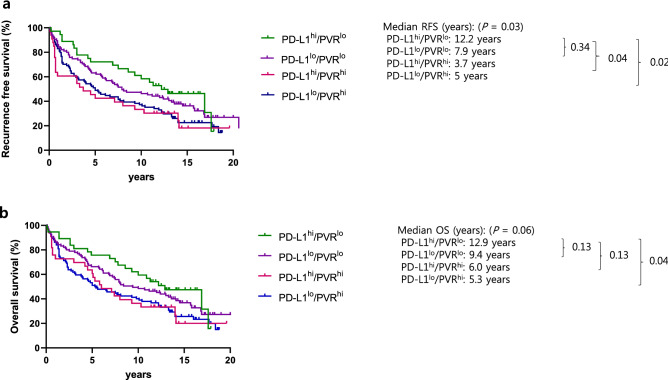
Kaplan–Meier analysis of (**a**) recurrence-free survival (RFS) and (**b**) overall survival (OS) by poliovirus receptor (PVR) and programmed death ligand-1 (PD-L1) expression. Patients were subdivided into four groups depending on their PD-L1 and PVR expression: (1) PD-L1^hi^/PVR^lo^, (2) PD-L1^lo^/PVR^lo^, (3) PD-L1^hi^/PVR^hi^, and (4) PD-L1^lo^/PVR^hi^.

The comparison of mRFS and mOS between these subgroups (depicted in Fig. 4) showed that the PD-L1^hi^/PVR^hi^ group had the shortest mRFS of 3.7 years, followed by the PD-L1^lo^/PVR^hi^, PD-L1^lo^/PVR^lo^, and PD-L1^hi^/PVR^lo^ groups (*P* = 0.03). The PD-L1^hi^/PVR^hi^ group had a tendency for the shortest mOS, although the result was not significant (*P* = 0.06). Subgroup analysis showed that the PD-L1^hi^/PVR^lo^ group had improved mOS compared to the PD-L1^lo^/PVR^hi^ group (HR: 2.43, 95% CI: 1.46–3.96; *P* = 0.04).

### Univariate and multivariate analyses of factors affecting survival

Univariate and multivariate analyses included factors such as age (< 65 *vs.* ≥ 65 years), sex, smoking status (non-smoker *vs.* smoker), cancer stage (I–II *vs.* III–IV), and PD-L1 and PVR status (PD-L1^hi^/PVR^lo^ *vs.* others). Other subgroups included the PD-L1^lo^/PVR^lo^, PD-L1^hi^/PVR^hi^, and PD-L1^lo^/PVR^hi^ groups.

Univariate analyses of RFS revealed that age ≥ 65 years (HR: 0.57, 95% CI: 0.43–0.77; *P* = 0.01), smoking history (HR: 0.63, 95% CI: 0.40–0.99; *P* = 0.04), advanced stage (HR: 0.46, 95% CI: 0.33–0.63; *P* = 0.01), and PVR > 20 (HR: 0.66, 95% CI: 0.50–0.89; *P* = 0.01) were associated with worse outcomes (see Supplementary Table S2 online). With the exception of smoking status, these factors were also significant in multivariate analyses. Similarly, age ≥ 65 years (HR: 0.57, 95% CI: 0.42–0.77; *P* = 0.01), advanced stage (HR: 0.41, 95% CI: 0.30–0.57; *P* = 0.01), and PVR > 20 (HR: 0.68, 95% CI: 0.50–0.91; *P* = 0.01) were associated with worse OS. On multivariate analyses, we found that all these factors remained significant as independent factors (see Supplementary Table S3 online). Overall, RFS and OS of surgically resected SQCC were affected by age, stage, and PVR expression. Of note, PD-L1 was not a prognostic factor for both RFS and OS in univariate and multivariate analyses. In contrast, high PVR expression was associated with short RFS and OS.

## Discussion

In our study, we found that the PD-L1 and PVR clusters were independently expressed as per TCGA RNA-sequencing data. In our cohort, PD-L1^hi^/PVR^hi^ status was correlated with the worst RFS, signifying that both PD-L1 and PVR are important factors for the recurrence of surgically resected SQCC. Similarly, PD-L1^hi^/PVR^hi^ status was also correlated with the shortest OS, although the result was not statistically significant. Subgroup analysis also showed that the PD-L1^lo^/PVR^hi^ group had shorter OS than the PD-L1^hi^/PVR^lo^ group, emphasizing the importance of PVR for OS, regardless of PD-L1 expression. Furthermore, multivariate analysis showed that high level of PVR expression was associated poor RFS and OS However, PD-L1 expression levels did not affect RFS or OS.

Currently, immunohistochemistry for PD-L1 is used to identify patients most likely to respond to immunotherapeutic agents, such as anti-PD-1 or anti-PD-L1 agents^24^. PD-L1 signaling blocks the T-cell response necessary for cytokine secretion and regulates the antitumor response by inducing regulatory T-cell differentiation^25,26^. Although targeting the PD-1/PD-L1 pathway with anti-PD-1 agents has shown reliable and robust results in metastatic and recurrent SQCC, some patients are unresponsive to these treatments^8–10^. Therefore, there is an unmet need to effectively treat non-responders.

Recently, a phase 2 trial of administering 2 cycles of atezolizumab, an anti-PD-L1 monoclonal antibody in neoadjuvant setting for resectable stage IB-IIIB NSCLC, yielded clinically meaningful major pathologic response rate of 20%^27^. The role of PD-L1 as well as PVR in neoadjuvant setting is yet to be elucidated.

Accumulating evidence suggests that targeting single checkpoints, such as the PD-1/PD-L1 pathway is insufficient for eliciting an immune response^28,29^. A better understanding of T-cell activity in combination with other checkpoint inhibitors may help overcome the lack of response due to the inhibition of immune activation. One potential mechanism is to block TIGIT, which interacts with PVR^30^. PVR binds to TIGIT, which is highly expressed in natural killer cells, T-cells, and regulatory T-cells, and induces immune escape of tumor cells^31,32^. Studies using mouse models have shown that the TIGIT/PVR axis is associated with inhibition of the proliferation, differentiation, and activation of T-cells and the activation of inhibitory T-cells (regulatory T-cells)^13,33^. Thus, targeting the TIGIT/PVR pathway may be a potential mechanism to overcome resistance to checkpoint inhibitors. Tiragolumab, an anti-TIGIT antibody, is a promising agent, which can be combined with atezolizumab to treat naïve, PD-L1-positive NSCLC.

There are few limitations to our study. First, our study was conducted retrospectively in a single center. The sole inclusion of Asian patients and the retrospective study design further limited the generalizability of our results. Validation using larger, prospective cohorts is warranted to bolster our findings. Second, our study lacked patients treated with immunotherapy after a systemic relapse, preventing the evaluation of the predictive roles of both PD-L1 and PVR. Identifying these factors in patients treated with immunotherapy in metastatic SQCC settings may help select those who may benefit from a combination of immunotherapeutic agents. Third, PD-L1 may possibly be underrepresented in some of the archival FFPE tissue blocks that date back to 1998. Indeed, studies have addressed that fresh biopsy samples better represent PD-L1 status^34,35^. However, PD-L1 was not a prerequisite at the time of tissue collection for this cohort, and this study adequately represents long term follow-up data on RFS and OS in surgically resected SQCC. Studies with more recent tissue samples are needed to validate our findings.

To our knowledge, the prognostic role of PD-L1 in correlation with PVR in SQCC has not been validated in any other study. Our study focused on the independent expression of PD-L1 and PVR and showed that patients with both high PD-L1 and PVR expression had a short RFS. Since patients with surgically resected SQCC often experience both local and distant relapses, better systemic treatment options for recurrent SQCC should be available in the immunotherapy era^5^.

In conclusion, overexpression of PVR is associated with poor prognosis in terms of RFS in surgically resected SQCC. Simultaneously targeting PD-L1 and the TIGIT/PVR axis with combination treatments may help overcome the lack of treatment response to immune checkpoint monotherapy.

## Methods

### Ethics declarations

The study was conducted in accordance with the Declaration of Helsinki. All patients provided written informed consent. The study was approved by the Institutional Review Board of Yonsei Cancer Center (approval number: 4–2018-1161).

### Expression of immune checkpoint ligand and receptor genes

We retrieved data on SQCC from TCGA to conduct a correlative analysis of ICLs and immune checkpoint receptors^23^. We collected data of normalized fragments per kilobase of transcript per million mapped reads for analysis of the RNA-sequencing dataset and performed a correlation analysis of 26 ICLs using Spearman’s correlation^36^. Using the hierarchical clustering method, we identified clusters of ICLs with strong co-expression patterns and selected two clusters that did not show correlations. Each ICL was categorized based on high (≥ 50th percentile) or low (< 50th percentile) expression of the target gene. The Kaplan–Meier method was used to calculate the OS, and Cox regression analysis was used for multivariate analysis.

### Patient selection

Data of 259 patients diagnosed with SQCC at the Yonsei Cancer Center between January 1998 and July 2020 were collected. The inclusion criteria for patient selection were surgically resected SQCC, archival tumor samples available for PVR and PD-L1 analysis, and availability of clinical data. Clinicopathological variables, such as age, sex, stage, pleural involvement, lymphovascular invasion, mutations in the *EGFR* and *KRAS* genes, smoking status, and adjuvant treatment, were evaluated. The American Joint Committee on Cancer (seventh edition) guidelines for tumor–node–metastasis classification were used to determine cancer stage^37^. An experienced pathologist (HSS) blinded to the clinical data reviewed and interpreted the pathology findings.

### Tissue microarray

We selected two or three different areas per sample representing tumor areas and developed tissue microarrays. Using formalin-fixed paraffin-embedded blocks, we collected tissue cores (3 mm in diameter) and arranged them in recipient paraffin blocks using a trephine. All tissue microarray blocks contained tumor lesions representing > 50% of the core, based on hematoxylin–eosin staining.

### Immunohistochemistry

A Ventana Benchmark XT autostainer (Ventana Medical Systems, Tucson, AZ, USA) was used to perform immunohistochemistry on 4-µm tissue microarray sections. The sections were stained with an anti–PD-L1 (clone SP263, Ventana Medical Systems) rabbit monoclonal primary antibody using the OptiView DAB IHC Detection kit for the PD-L1 IHC assay^38^. PVR antibody (rabbit monoclonal, cloneD8A5G, Cell Signaling Technology) was diluted to 1:100, treated, and incubated at 37 °C for 32 min for the PVR IHC assay. Using ultraview universal DAB Detection Kit (Ventana Medical Systems), the signals for PD-L1 and PVR were detected. Both the PD-L1 and PVR scores were interpreted as TPS according to the methods described in previous studies^9^.

PD-L1 high and low expression status were determined according to the median cut-off value of 5% staining of tumor cells (see Supplementary Fig. S1 online). PVR expression was classified as high (> 20% TPS) or low (≤ 20% TPS) based on the median value (see Supplementary Fig. S1 online). Patients were then categorized into four groups depending on PD-L1 and PVR expression: PD-L1^hi^/PVR^lo^, PD-L1^lo^/PVR^lo^, PD-L1^hi^/PVR^hi^, and PD-L1^lo^/PVR^hi^ (Fig. 1a–d).

For the CD8^+^ analysis, the percentages of CD8 + lymphocytes (RTU, clone C8/144B (Dako, Glostrup, Denmark) compared with the total amount of nucleated cells in the stromal compartments were assessed^39^. CD8^+^ expression was also determined as high (≥ 10) or low (< 10) based on the median cutoff value of 10%.

### Statistical analysis

The correlations between variables were analyzed using Fischer’s exact test for categorical variables and the sample *t*-test for continuous variables. We used the Kaplan–Meier method to calculate OS and RFS. RFS was defined as the time from the date of surgery to the date of recurrence, last follow-up, or death. OS was defined as the time between the date of diagnosis and the date of last follow-up or death. Cox regression with backward elimination method was used for multivariate analysis of OS. Statistical analyses were two-sided with *P* = 0.05 as the level of significance. All statistical analyses were performed using Statistical Package for the Social Sciences (version 25; IBM, Chicago, IL, USA) and GraphPad Prism 8.0 Software (GraphPad Software, Inc., San Diego, CA).

## Supplementary Information

Supplementary Information 1.

Supplementary Information 2.

Supplementary Information 3.

Supplementary Information 4.

Supplementary Information 5.

## Data Availability

The datasets used and/or analyzed during the current study are available from the corresponding author on reasonable request.
