# Publisher Correction: Overexpression of PVR and PD‑L1 and its association with prognosis in surgically resected squamous cell lung carcinoma

**DOI:** 10.1038/s41598-021-94253-x

**Published:** 2021-07-26

**Authors:** Jii Bum Lee, Min Hee Hong, Seong Yong Park, Sehyun Chae, Daehee Hwang, Sang‑Jun Ha, Hyo Sup Shim, Hye Ryun Kim

**Affiliations:** 1 Division of Medical Oncology, Department of Internal Medicine, Yonsei Cancer Center, Yonsei University College of Medicine, 50‑1 Yonsei‑ro, Seodaemun‑gu, Seoul, Republic of Korea; 2 Division of Hemato-oncology, Wonju Severance Christian Hospital , Yonsei University College of Medicine, Wonju, Republic of Korea; 3 Department of Thoracic and Cardiovascular Surgery, Yonsei University College of Medicine, Seoul, Korea; 4 Korea Brain Bank, Korea Brain Research Institute, Daegu, Korea; 5 Department of Biological Sciences, Seoul National University, Seoul, Korea; 6 Department of Biochemistry, College of Life Science & Biotechnology, Yonsei University, Seoul, Korea; 7 Department of Pathology, Yonsei University College of Medicine, 50‑1 Yonsei‑ro, Seodaemun‑gu, Seoul, Republic of Korea

Correction to: *Scientific Reports* 10.1038/s41598-021-87624-x, published online 20 April 2021

The Funding section in the original version of this Article was incomplete.

“This research was supported by the Basic Science Research Program through the National Research Foundation of Korea (NRF) funded by the Ministry of Education (NRF-2018R1D1A1B07047811, to HSS).”

now reads:

“This work was supported by National Research Foundation of Korea (NRF) grants funded by the Korean Government (MSIT) (NRF 2017M3A9E9072669, 2017M3A9E8029717, and NRF-2019M3A9B6065231(sc) awarded to HRK). This research was supported by the Basic Science Research Program through the National Research Foundation of Korea (NRF) funded by the Ministry of Education (NRF-2018R1D1A1B07047811, to HSS).”

The original Article has been corrected.

