# Supplementary figures and images for: Overexpression of PVR and PD-L1 and its association with prognosis in surgically resected squamous cell lung carcinoma
Source: Sci Rep. 2021 Apr 20;11:8551. doi: 10.1038/s41598-021-87624-x (PMC8058057; doi:10.1038/s41598-021-87624-x)

## Slide 1
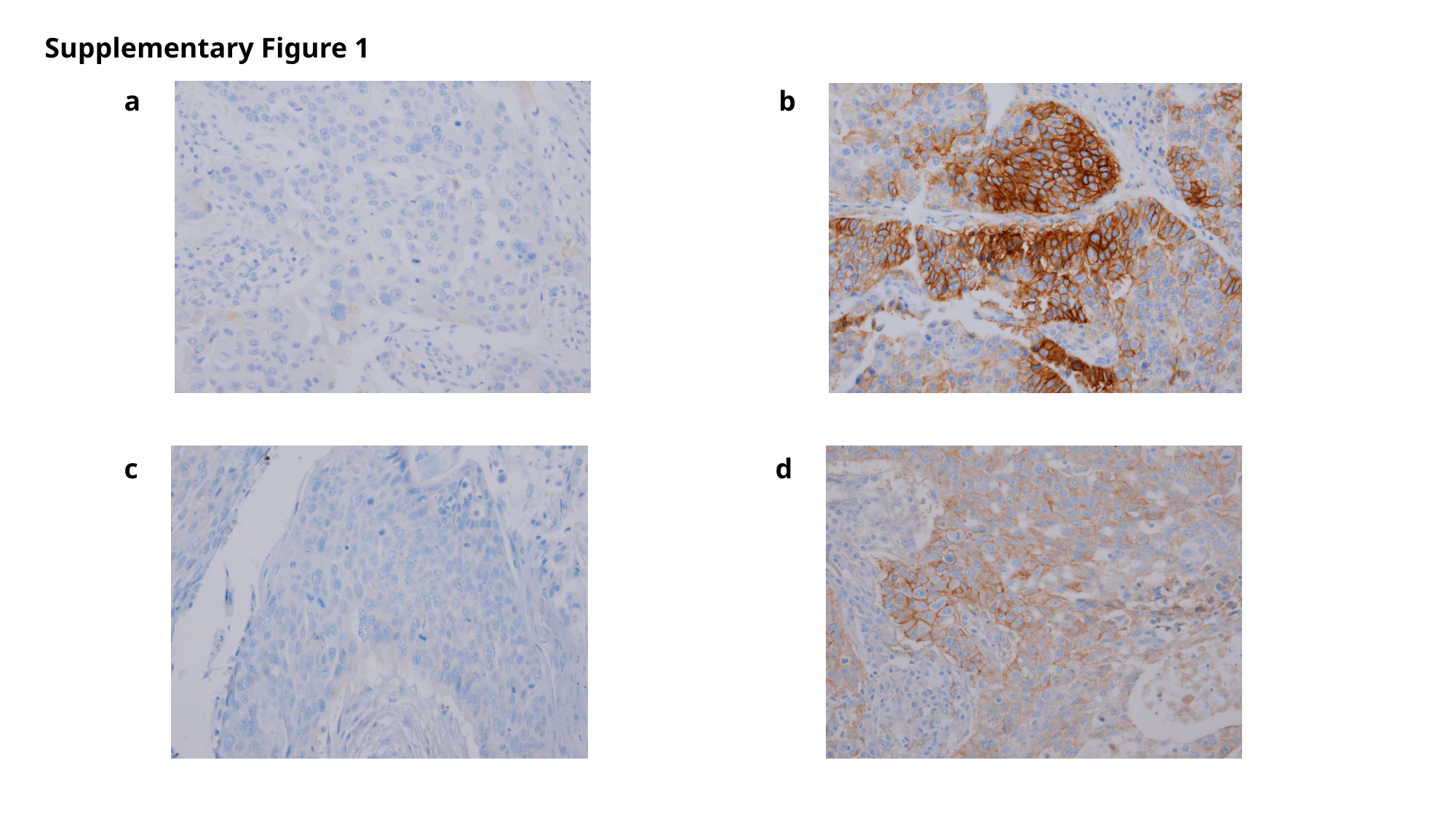

Supplementary Figure 1
a
b
c
d

Supplement: Supplementary file 1 — Supplementary Information 1. [file 41598_2021_87624_MOESM1_ESM.pptx]

## Slide 1
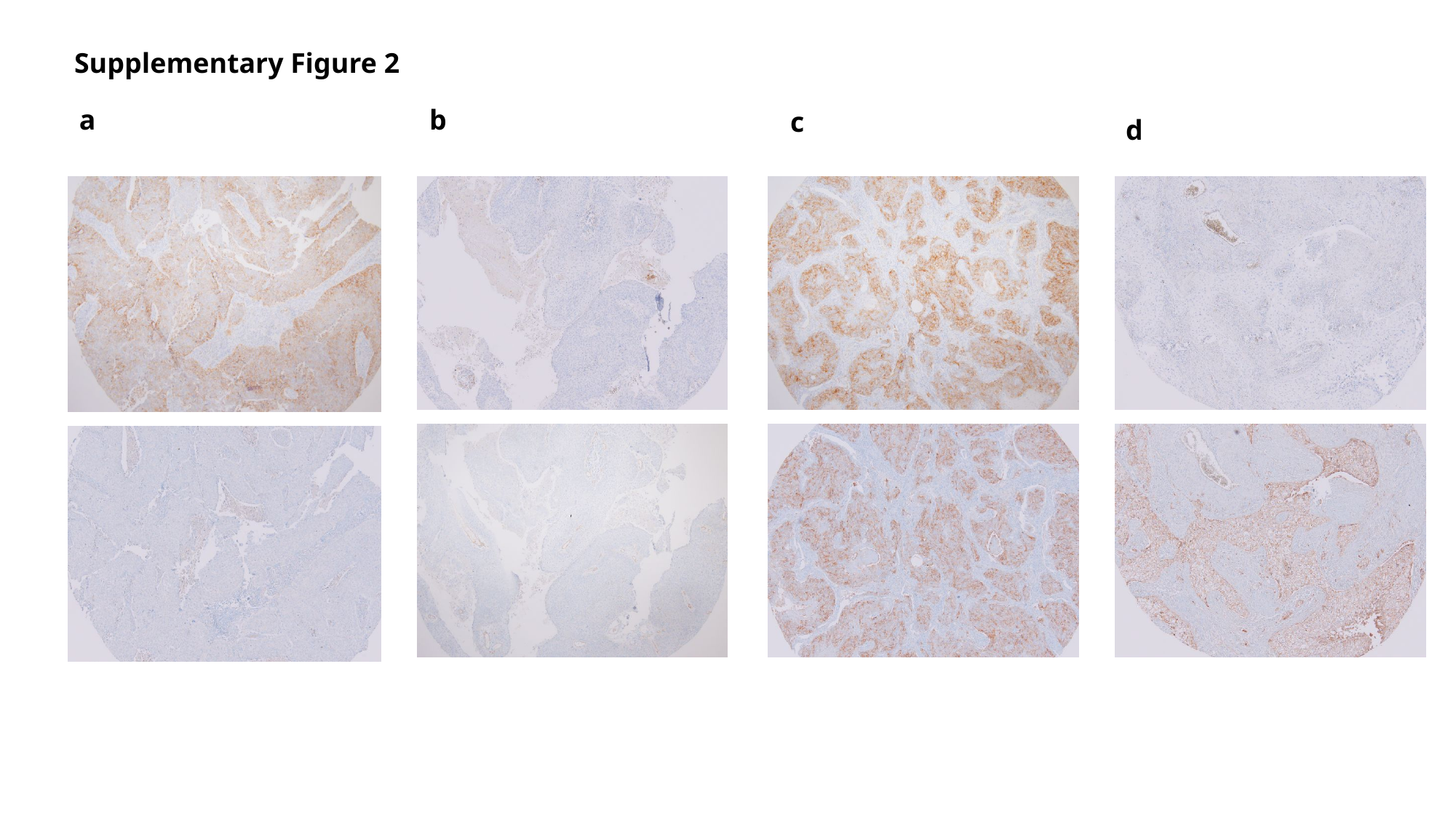

Supplementary Figure 2
a
b
c
d

Supplement: Supplementary file 2 — Supplementary Information 2. [file 41598_2021_87624_MOESM2_ESM.pptx]

## Slide 1
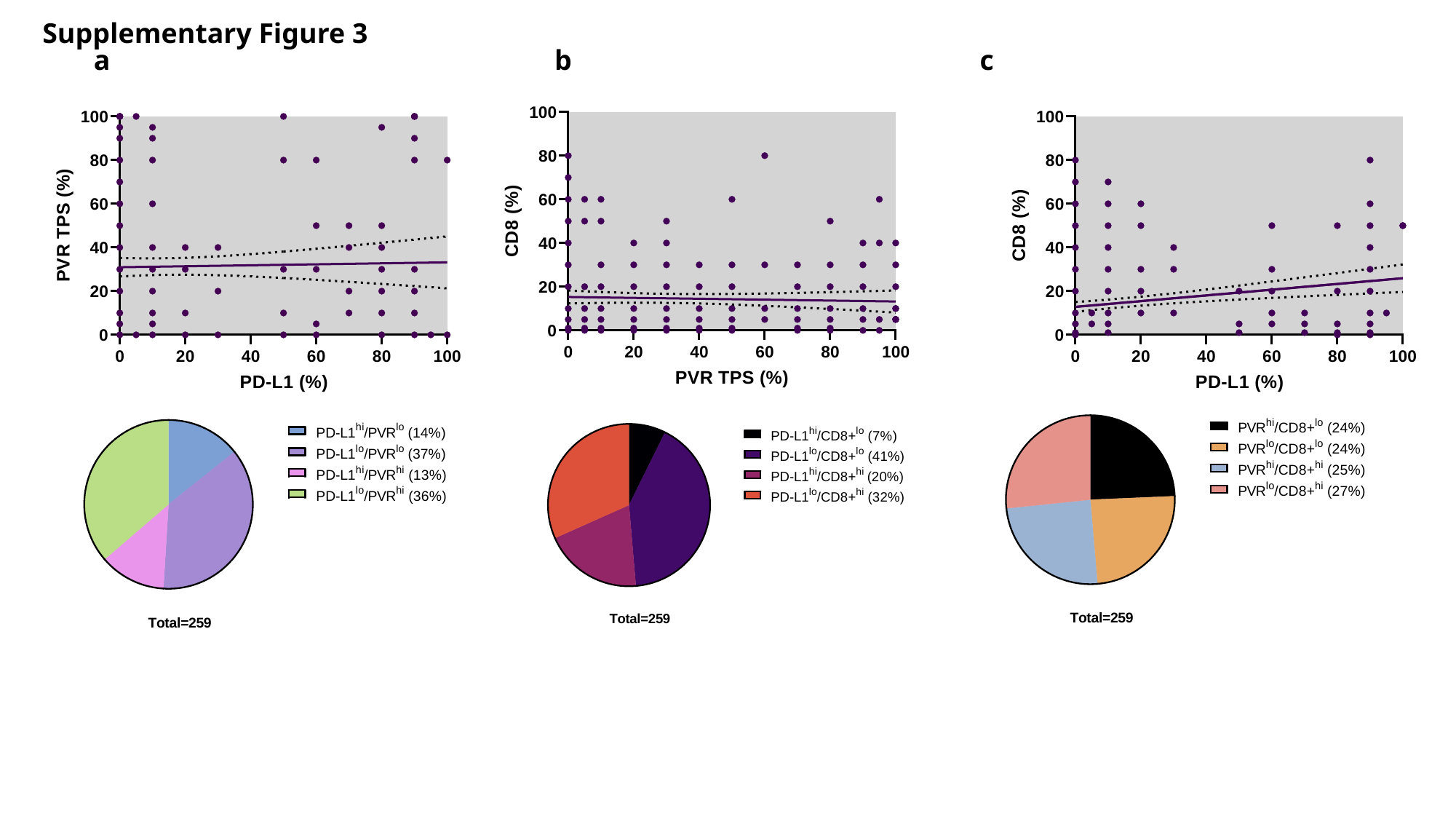

Supplementary Figure 3
a
b
c

Supplement: Supplementary file 3 — Supplementary Information 3. [file 41598_2021_87624_MOESM3_ESM.pptx]

## Slide 1
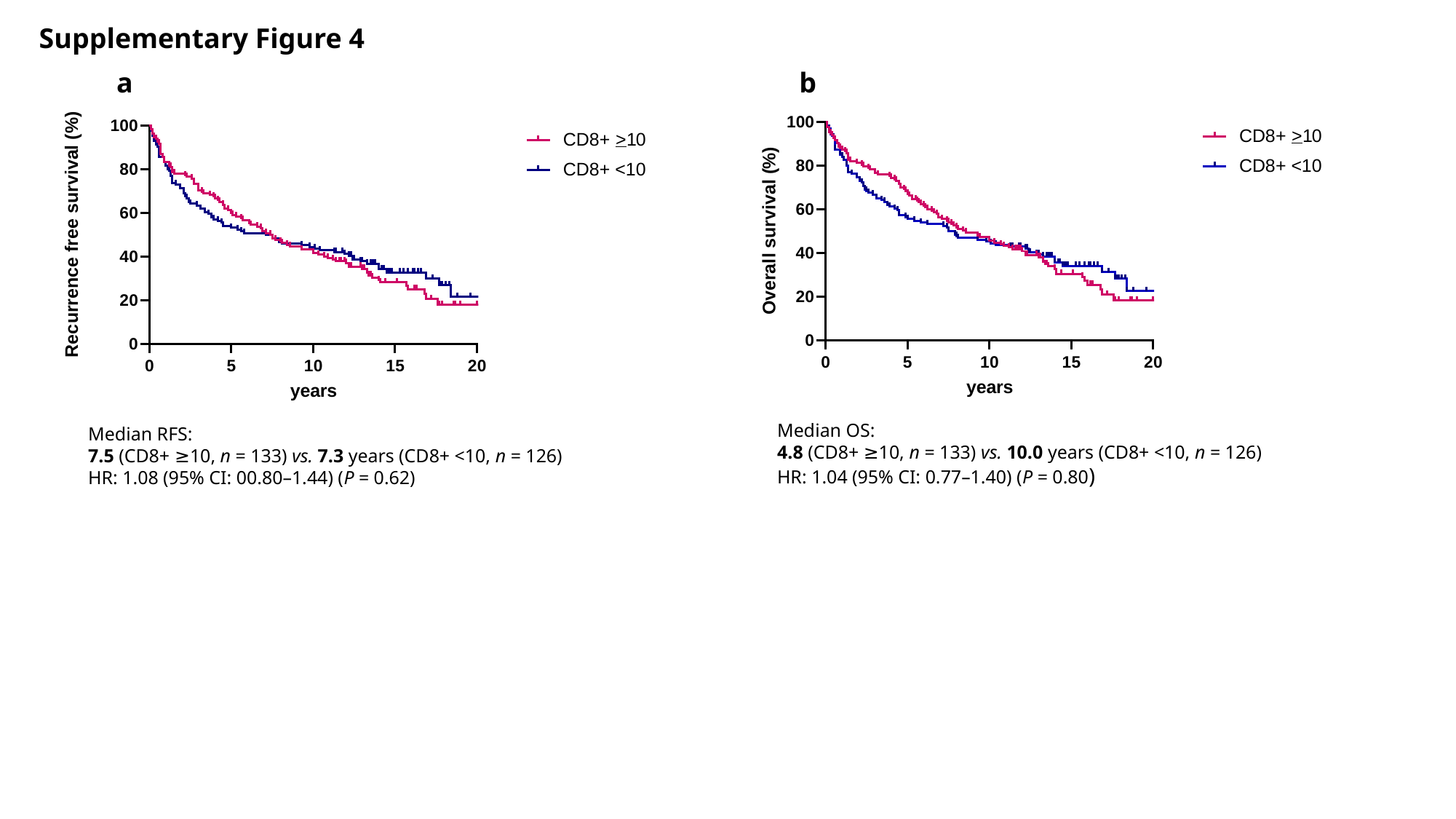

Supplementary Figure 4
a
b
Median OS:
4.8 (CD8+ ≥10, n = 133) vs. 10.0 years (CD8+ <10, n = 126)
HR: 1.04 (95% CI: 0.77–1.40) (P = 0.80)
Median RFS:
7.5 (CD8+ ≥10, n = 133) vs. 7.3 years (CD8+ <10, n = 126)
HR: 1.08 (95% CI: 00.80–1.44) (P = 0.62)

Supplement: Supplementary file 4 — Supplementary Information 4. [file 41598_2021_87624_MOESM4_ESM.pptx]
