# Supplementary material for: Overexpression of PVR and PD-L1 and its association with prognosis in surgically resected squamous cell lung carcinoma
Source: Sci Rep. 2021 Apr 20;11:8551. doi: 10.1038/s41598-021-87624-x (PMC8058057; doi:10.1038/s41598-021-87624-x)
Supplement: Supplementary file 5 — Supplementary Information 5. [file 41598_2021_87624_MOESM5_ESM.docx]

**Overexpression of PVR and PD-L1 and its association with prognosis in surgically resected squamous cell lung carcinoma**

Jii Bum Lee, Min Hee Hong, Seong Yong Park, Sehyun Chae, Daehee Hwang, Sang-Jun Ha, Hyo Sup Shim, and Hye Ryun Kim

**Supplementary Fig. S1.** Programmed death ligand-1 (PD-L1) and poliovirus receptor (PVR) expression on immunohistochemistry with 200× magnification. (**a**) PD-L1-negative (tumor proportion score [TPS] <5%) and (**b**) PD-L1-positive (TPS ≥5%). (**c**) PVR^lo^ (≤20) and (**d**) PVR^hi^ (>20).

**Supplementary Fig. S2.** Programmed death ligand-1 (PD-L1) and poliovirus receptor (PVR) expression on immunohistochemistry with 40× magnification. (**a**) PD-L1^hi^/PVR^lo^, (**b**) PD-L1^lo^/PVR^lo^, (**c**) PD-L1^hi^/PVR^hi^, and (**d**) PD-L1^lo^/PVR^hi^.

**Supplementary Fig. S3.** Pie chart of the distribution of four groups by the expression of (**a**) programmed death ligand-1 (PD-L1) and poliovirus receptor (PVR), (**b**) PD-L1 and CD8+, and (**c**) PVR and CD8+. TPS, tumor proportion score.

**Supplementary Fig. S4.** Kaplan–Meier analysis of (**a**) recurrence-free survival (RFS) and (**b**) overall survival (OS) by CD8+ expression. CI, confidence interval; HR, hazard ratio.

**Supplementary Table S1.** Baseline characteristics of patients by PVR and PD-L1

| **Clinical Characteristics** | **All patients** | | **PVR high** | | **PVR low** | | **PD-L1 high** | | **PD-L1 low** | |
| --- | --- | --- | --- | --- | --- | --- | --- | --- | --- | --- |
|  | **No.** | **%** | **No.** | **%** | **No.** | **%** | **No.** | **%** | **No.** | **%** |
| **No. of patients** | 259 | 100 | 127 | 49 | 132 | 51 | 70 | 27 | 189 | 73 |
| **Age, years** |  |  |  |  |  |  |  |  |  |  |
| <65 years | 123 | 47 | 60 | 47 | 63 | 48 | 33 | 47 | 90 | 48 |
| >65 years | 136 | 53 | 67 | 53 | 69 | 52 | 37 | 53 | 99 | 52 |
| **Sex** |  |  |  |  |  |  |  |  |  |  |
| Male | 241 | 93 | 119 | 94 | 122 | 92 | 66 | 94 | 175 | 93 |
| Female | 18 | 7 | 8 | 6 | 10 | 8 | 4 | 6 | 14 | 7 |
| **pT stage** |  |  |  |  |  |  |  |  |  |  |
| T1 | 35 | 14 | 14 | 11 | 21 | 16 | 7 | 10 | 28 | 15 |
| T2 | 175 | 67 | 83 | 65 | 92 | 70 | 50 | 71 | 125 | 66 |
| T3 | 41 | 16 | 25 | 20 | 16 | 12 | 11 | 16 | 30 | 16 |
| T4 | 8 | 3 | 5 | 4 | 3 | 2 | 2 | 3 | 6 | 3 |
| **pN stage** |  |  |  |  |  |  |  |  |  |  |
| N0 | 142 | 55 | 68 | 53 | 74 | 56 | 38 | 54 | 104 | 55 |
| N1 | 71 | 27 | 34 | 27 | 37 | 28 | 20 | 29 | 51 | 27 |
| N2 | 44 | 17 | 24 | 19 | 20 | 15 | 12 | 17 | 32 | 17 |
| N3 | 2 | 1 | 1 | 1 | 1 | 1 | 0 | 0 | 2 | 1 |
| **AJCC stage (7th)** |  |  |  |  |  |  |  |  |  |  |
| I | 122 | 47 | 58 | 46 | 64 | 48 | 31 | 45 | 91 | 48 |
| II | 76 | 29 | 33 | 26 | 43 | 32 | 24 | 34 | 52 | 28 |
| IIIA | 54 | 21 | 32 | 25 | 22 | 17 | 12 | 17 | 42 | 22 |
| IIIB | 6 | 2 | 4 | 3 | 2 | 2 | 2 | 3 | 4 | 2 |
| IV | 1 | 1 | 0 | 0 | 1 | 1 | 1 | 1 | 0 | 0 |
| **Pleural involvement** |  |  |  |  |  |  |  |  |  |  |
| Positive | 68 | 26 | 37 | 29 | 31 | 23 | 16 | 23 | 52 | 28 |
| Negative | 191 | 74 | 90 | 71 | 101 | 77 | 54 | 77 | 137 | 72 |
| **Lymphovascular invasion** |  |  |  |  |  |  |  |  |  |  |
| Positive | 28 | 11 | 16 | 13 | 12 | 9 | 8 | 11 | 20 | 11 |
| Negative | 231 | 89 | 111 | 87 | 120 | 91 | 62 | 89 | 169 | 89 |
| **EGFR mutation** |  |  |  |  |  |  |  |  |  |  |
| Yes | 4 | 2 | 2 | 2 | 2 | 2 | 0 | 0 | 4 | 2 |
| No | 68 | 26 | 31 | 24 | 37 | 28 | 32 | 46 | 36 | 19 |
| Not done | 187 | 72 | 94 | 74 | 93 | 70 | 38 | 54 | 149 | 79 |
| **KRAS mutation** |  |  |  |  |  |  |  |  |  |  |
| Yes | 0 | 0 | 0 | 0 | 0 | 0 | 0 | 0 | 0 | 0 |
| No | 68 | 26 | 32 | 25 | 36 | 27 | 29 | 41 | 39 | 21 |
| Not done | 191 | 74 | 95 | 75 | 96 | 73 | 41 | 59 | 150 | 79 |
| **Smoking status** |  |  |  |  |  |  |  |  |  |  |
| Never-smoker | 39 | 15 | 17 | 13 | 22 | 17 | 9 | 13 | 30 | 16 |
| Former-smoker | 114 | 44 | 52 | 41 | 62 | 47 | 30 | 43 | 84 | 44 |
| Current-smoker | 106 | 41 | 58 | 46 | 48 | 36 | 31 | 44 | 75 | 40 |
| **Adjuvant therapy** |  |  |  |  |  |  |  |  |  |  |
| **Adjuvant chemotherapy** |  |  |  |  |  |  |  |  |  |  |
| Yes | 221 | 85 | 110 | 87 | 111 | 84 | 62 | 89 | 159 | 84 |
| No | 38 | 15 | 17 | 13 | 21 | 16 | 8 | 11 | 30 | 16 |
| **Adjuvant radiotherapy** |  |  |  |  |  |  |  |  |  |  |
| Yes | 44 | 17 | 25 | 20 | 19 | 14 | 10 | 14 | 34 | 18 |
| No | 215 | 83 | 102 | 80 | 113 | 86 | 60 | 86 | 155 | 82 |

PD-L1, programmed death ligand-1; PVR, poliovirus receptor ; AJCC, American Joint Committee on Cancer; EGFR, epidermal growth factor receptor

**Supplementary Table S2.** Univariate and multivariate analyses of recurrence-free survival

| **Variable** | **Category** | **Univariate analysis** | | | **Multivariate analysis** | | |
| --- | --- | --- | --- | --- | --- | --- | --- |
|  |  | **HR** | **95% CI** | ***P*-value** | **HR** | **95% CI** | ***P*-value** |
| Age (years) | <65 *vs.* ≥65 | **0.57** | **0.43–0.77** | **0.01** | **1.82** | **1.35–2.45** | **0.01** |
| Sex | Male *vs.* female | 0.87 | 0.92**–**3.58 | 0.08 | — | — | — |
| Smoking status | Non-smoker *vs.* smoker | **0.63** | **0.40–0.99** | **0.04** | — | — | — |
| Stage | Stage I–II *vs.* III–IV | **0.46** | **0.33–0.63** | **0.01** | **2.16** | **1.56–2.99** | **0.01** |
| CD8^+^ | CD8^+^<10% *vs.* CD8^+^>10% | 0.92 | 0.69**–**1.23 | 0.58 | — | — | — |
| PVR | PVR ≤20 *vs.* PVR >20 | **0.66** | **0.50–0.89** | **0.01** | **1.35** | **1.01–1.82** | **0.045** |
| PD-L1 | TPS <5% *vs.* TPS ≥5% | 1.06 | 0.76**–**1.47 | 0.75 | — | — | — |

CI, confidence interval; HR, hazard ratio; PD-L1, programmed death ligand-1; PVR, poliovirus receptor; TPS, tumor proportion score

**Supplementary Table S3.** Univariate and multivariate analyses of overall survival

| **Variable** | **Category** | **Univariate analysis** | | | **Multivariate analysis** | | |
| --- | --- | --- | --- | --- | --- | --- | --- |
|  |  | **HR** | **95% CI** | ***P*-value** | **HR** | **95% CI** | ***P*-value** |
| Age (years) | <65 *vs.* ≥65 | **0.57** | **0.42–0.77** | **0.01** | **1.81** | **1.33–2.45** | **0.01** |
| Sex | Male *vs.* female | 1.71 | 0.87**–**3.38 | 0.12 | — | — | — |
| Smoking status | Non-smoker *vs.* smoker | 1.58 | 1.00**–**2.49 | 0.05 | — | — | — |
| Stage | Stage I–II *vs.* III–IV | **0.41** | **0.30–0.57** | **0.01** | **2.42** | **1.74–3.36** | **0.01** |
| CD8^+^ | CD8^+^<10% *vs*. CD8^+^>10% | 0.97 | 0.72**–**1.30 | 0.81 | — | — | — |
| PVR | PVR ≤20 *vs.* PVR >20 | **0.68** | **0.50–0.91** | **0.01** | **1.36** | **1.01–1.83** | **0.044** |
| PD-L1 | PD-L1 <5% *vs.* PD-L1 ≥5% | 1.12 | 0.80**–**1.57 | 0.51 | — | — | — |

CI, confidence interval; HR, hazard ratio; PVR, poliovirus receptor; PD-L1, programmed death ligand-1; TPS, tumor proportion score
